# Clinical results and motion analysis following arthroscopic anterior stabilization of the shoulder using bioknotless anchors

**DOI:** 10.4103/0973-6042.70821

**Published:** 2010

**Authors:** Stephen Cooke, Owain Ennis, Haroon Majeed, Aziz Rahmatalla, Vinod Kathuria, Roger Wade

**Affiliations:** University Hospital North Staffordshire, Princes Road, Stoke-on-Trent, Staffordshire, ST4 7LN, UK; 1 Bionics Laboratory, University Hospital North Staffordshire, Princes Road, Stoke-on-Trent, Staffordshire, ST4 7LN, UK; 2 Stafford General Hospital, Weston Road, Stafford, ST16 3SA, UK

**Keywords:** Arthroscopic stabilization, motion analysis, shoulder dislocation, shoulder instability, suture anchors

## Abstract

**Purpose:**

Traumatic anterior dislocation of the shoulder is a common occurrence increasingly being treated arthroscopically. This study aims to determine the outcome of arthroscopic anterior stabilization using bioknotless anchors and analyze the motion in a subset of these patients.

**Materials and Methods:**

The outcome of 20 patients who underwent arthroscopic anterior stabilization using the bioknotless system was studied (average follow-up 26 months). Four of these patients underwent motion analysis of their shoulder pre- and post-operatively.

**Results:**

15% were dissatisfied following surgery and the recurrence of instability was also 15%. Those who were dissatisfied or suffered recurrent symptoms had statistically significant lower constant scores at the final follow up. Pre-operative motion analysis showed a disordered rhythm of shoulder rotation which was corrected following surgery with minimal loss of range of motion.

**Conclusions:**

Our success rate was comparable to similar arthroscopic techniques and results published in the literature. Patient satisfaction depended more on return to usual activities than recurrence of symptoms. There was very little reduction in range of movement following surgery and the rhythm of shoulder motion, particularly external rotation in abduction was improved.

**Level of Evidence:**

Four retrospective series.

## INTRODUCTION

**Figure 1:**
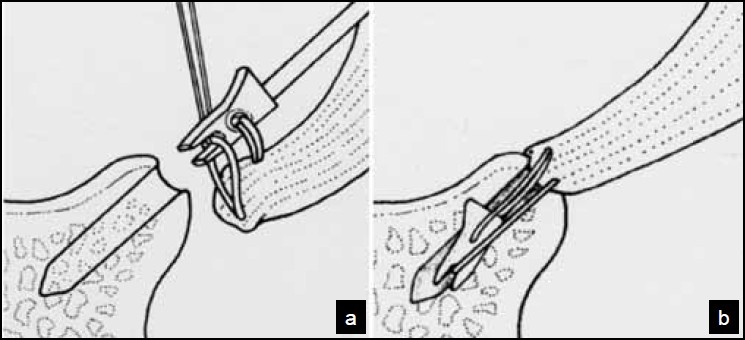
Diagram illustrating the bioknotless system. (a) The utility loop has pulled the ethibond suture through the capsulolabral complex which is then grasped between the teeth of the anchor (b) The anchor is then buried in the predrilled hole in the glenoid rim ensuring good tension is achieved.

Traumatic anterior dislocation of the shoulder is a very common condition, with an approximate incidence of 10-20 per 100,000 per year.[1] Following the initial event there is a high risk of recurrence, estimates between 20% and 90% have been reported in the literature[2–5] with approximately 17% occurring in the first week and the remainder up to 7 years later.[6] Arthroscopic anterior stabilization is a well recognized and increasingly popular technique with level 1 evidence to support its use in this condition.[7] A variety of labral fixation devices have been described. We present our experience using the bioknotless anchor (DePuy Mitek, Raynham, MA) [Figure 1] and use the fastrack motion analysis system (Polhemus, Colchester, VT) in a small cohort of these patients to assess pre- and post-operative shoulder kinematics.

## MATERIALS AND METHODS

Between October 2004 and April 2006, 20 shoulders in 20 patients underwent arthroscopic anterior stabilization of the shoulder using bioknotless anchors. All patients were assessed pre-operatively and operated on by the two senior authors (VK and RW). There were 16 males and 4 females with an average age of 28 (range 17 to 53). Patients had an average of four dislocations (minimum two) prior to surgery and were deemed to be suitable candidates for arthroscopic stabilization. All patients presented initially with traumatic anterior dislocation of the shoulder. Sixteen patients participated in recreational contact sports and three at a regional or national level. Ten had occupations involving heavy manual labor.

The fastrack system is a spatial tracking system that uses an electromagnetic field to determine the 3D position and orientation of markers in space. A biotechnologist (AR) placed the makers on the bony landmarks of the sternum (the manubiosternal joint), scapula (the lateral edge of the acromion and the inferior point of the scapular wing), humerus and elbow (medial and lateral epidondyles) and wrist (the ulnar head); a complete picture of shoulder range and rate of motion can be built. It has a fast sampling rate (30 Hz) and has been shown to be an effective and valid method for the assessment of upper limb motion.[8] Six patients underwent fastrack analysis of their shoulder movements as part of their pre-operative investigations. Four of these went on to require stabilization and are included in the study group. The other two patients did not have surgery. Five of these patients (four who had surgery and one who did not) had repeat fastrack analysis at the time of their latest post-operative review.

### Operative technique

**Figure 2:**
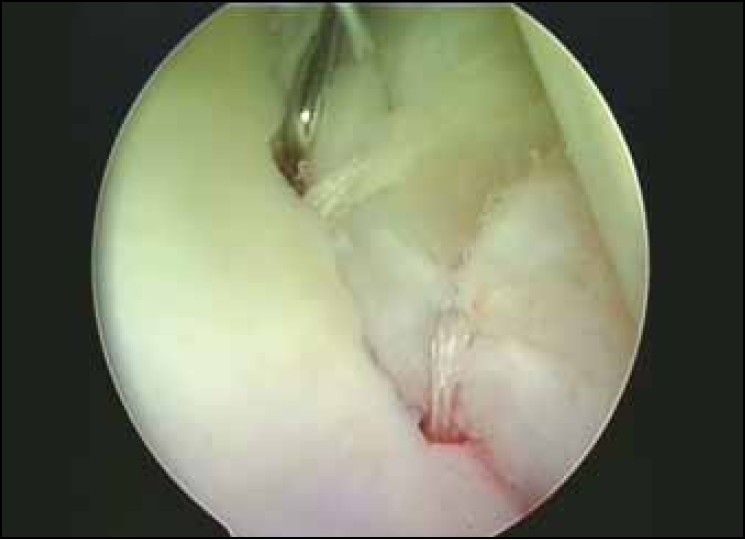
Three bioknotless anchors have been placed to secure a Bankart lesion (two of which can be seen in this view). The repair is probed to ensure adequate tension and stability have been achieved

Patients are placed in the beach chair position under general anesthetic and interscalene brachial plexus block. Examination under anesthesia is carried out followed by a standard diagnostic arthroscopy. All patients were found to have increased anterior translation of the humeral head and one had increased posterior translation in addition. All patients were found to have a Bankart lesion, seven of whom had an additional Hill-Sachs lesion and three also had SLAP lesions. Repair of the Bankart and/or SLAP lesion was performed using the bioknotless anchors as described elsewhere.[9,10] For small lesions, two anchors may suffice but for larger defects, especially when a Bankart extends into a SLAP lesion, four or five anchors may be required. In our series, on an average of 2.6 anchors were used (range 2-4) [Figure 2].

### Post-operative management

All patients were placed in a sling in internal rotation post-operatively. Once pain allowed, they were taught pendulum exercises and encouraged to perform these daily.

After 1 week they were reviewed by a physiotherapist who commenced passive range of movement exercises. Assisted active and active mobilization was introduced over the following 6 to 12 weeks. External rotation beyond neutral was avoided for 6 weeks and combined external rotation and abduction for 12 weeks. All patients were advised to avoid contact sports and overhead weight-bearing activities for 6 months.

Follow-up assessment was carried out by the first author and consisted of constant score evaluation in both affected and unaffected shoulders, patient satisfaction, and recurrence of instability or dislocation. Post-operative radiographs were not routinely obtained and were only done if clinically indicated. Fastrack analysis was carried out on those patients that had undergone pre-operative analysis. The average length of follow-up was 26 months (range 18 to 35).

## RESULTS

Average constant scores returned to 85%±7.7% of that measured in the contralateral arm [Table 1]. Fifteen patients had scores >75 and 5 had scores between 50 and 75. Three patients were unsatisfied with their surgery (15%). Only one of these three had recurrent symptoms, other the two stating that their dissatisfaction was due to their inability to return to pre-injury occupations or sports. Two patients re-dislocated (10%) and 1 had symptomatic instability (5%). One of the recurrent dislocations was due to a new traumatic event and the other occurred with minimal trauma. When all five patients who were either dissatisfied with their surgery or had recurrent symptoms (or both) were grouped together, and their constant scores averaged 76% ± 8.1% of the control arm. This compares with 88% ± 4.9% in the rest of the group. This was statistically significant (*P*<0.05, Student’s t-test).

**Table 1:** Details of each patient included in the study

Patient no.	Age	Pathology	No. of anchors	Constant score (operated limb)	Constant score (% of control limb)	Lateral elevation^a^	External rotation^b^	Post-op review
1	17	B,HS	4	84	88.4	151-180°	3	
2	19	B	2	50	66.7	91-120°	1	SI,U
3	19	B	2	82	91.1	151-180°	3	
4	21	B	3	80	84.2	151-180°	2	
5	21	B	2	81	84.4	151-180°	3	
6	21	B,HS	2	81	90.0	151-180°	3	
7	22	B	2	86	89.6	151-180°	3	
8	23	B	2	83	86.5	151-180°	3	
9	24	B,SLAP	3	86	93.5	151-180°	3	
10	24	B,HS	2	62	77.5	121-150°	1	D
11	25	B,HS	3	80	84.2	151-180°	3	D
12	26	B	2	73	84.0	151-180°	3	U
13	26	B,HS,SLAP	4	84	87.5	151-180°	3	
14	26	B	3	84	94.4	151-180°	3	
15	34	B,HS	3	72	78.3	121-150°	2	
16	38	B	2	76	81.7	121-150°	2	
17	38	B,HS	3	81	90.0	151-180°	3	
18	43	B	2	85	94.4	151-180°	3	
19	46	B,SLAP	3	87	93.5	151-180°	3	
20	53	B	2	61	69.3	91-120°	1	U

B: Bankart lesion, HS: Hill-Sachs lesion, SLAP: superior labrum anterior posterior tear, OA: osteoarthritis, SI: symptomatic instability, D: dislocation, U: patient unsatisfied.

a Lateral elevation as recorded by constant score assessment at final review.

b External rotation as recorded by Constant score assessment at final review, 1: hand behind head, elbow forward, 2: hand behind head, elbow back, 3: full

**Figure 3:**
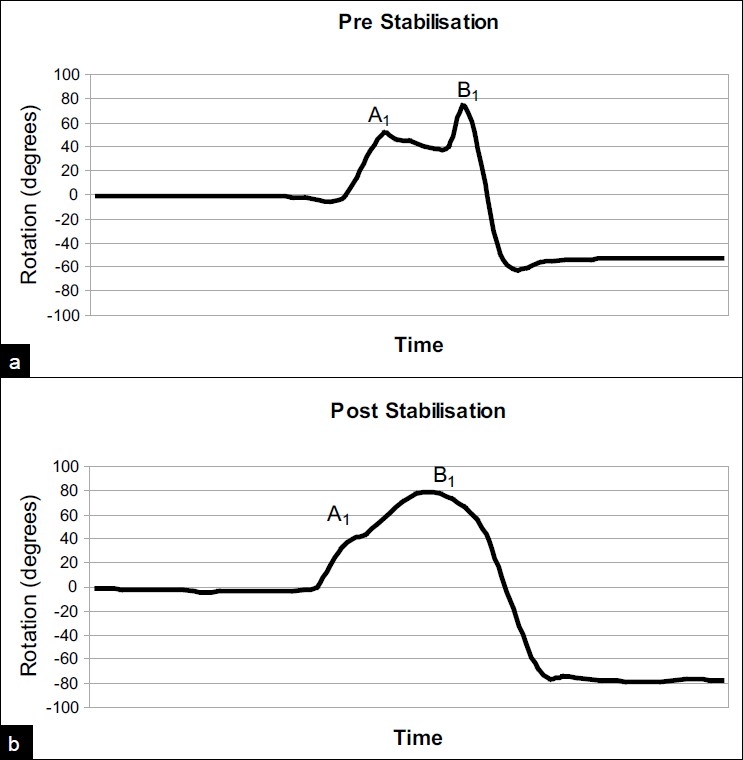
Fastrack analysis of shoulder rotation in 90° abduction in one patient (a) pre and (b) post operatively. The patient was asked to rotate from neutral to full external rotation (positive degrees) to full internal rotation (negative degrees). Point A1 shows the start of apprehension and B1 the limit of external rotation pre-operatively. The corresponding points post-operatively are indicated (A2 and B2)

Of the 16 sportsmen and women, 10 returned to their pre-injury level of participation and 7 of 10 patients returned to heavy physical occupations. Overall, 13 patients returned to their previous activities and 7 did not (35%). We did not encounter any other surgical complications. Fastrack analysis revealed a very slight reduction in range of motion post-operatively. External rotation in abduction measured in the four patients was reduced by 4° ± 4°. The range of motion correlated with that measured clinically. Dynamically, it was noted that the velocity of motion, represented by the gradient of the curve, was much more constant following surgery [Figure 3]. The reasons for this are postulated in the discussion.

## DISCUSSION

The open capsulolabral repair remains the gold standard against which other methods are measured. There is considerable evidence showing low recurrence in the order of 5-10%, with high patient satisfaction many years post-operatively.[11] There are concerns regarding functional loss of motion (particularly external rotation), however, as well as long recovery times with up to 1/3 of patients not returning to predislocation activities, particularly competitive sports.[11] Arthroscopic stabilization has developed in an attempt to minimize these problems and numerous methods have been described.

The knotless anchor, developed by Thal,[9] enables capsulolabral repair directly to the glenoid rim. As the name implies, the complexities of tying and tensioning arthroscopic knots are avoided and no bulky tie is left within the joint. The bioknotless anchor affords the same benefits as the knotless system but with an absorbable anchor, thus avoiding the reported complication of anchors backing out and damaging the articular surface.[12] The anchor absorbs over a long period of time retaining 90% of its strength at 2 months and 60% at 6 months. Complete absorption takes up to 4 years and osteolysis secondary to back out of anchors prior to resorption has been reported.[13] There have also been reports of foreign body reactions at the anchor site,[14] but this was not encountered in our series.

Our rates of re-dislocation and instability, 10% and 5%, respectively, are in broad agreement with other studies using the bioknotless anchor.[10,12] Shoulder function, as measured by the constant score returned to 85% of maximum (taken as that measured in the contralateral normal arm). As might be expected there were significantly better scores in those who were both satisfied with their surgery and had no recurrence of symptoms. More unexpectedly, of the three unsatisfied patients (15%), only one had recurrent instability (patient 2) and conversely two of the three patients with post-operative symptoms said they were satisfied with their surgery (patients 10 and 11). One of these had had a single post-op dislocation due to a new traumatic event (patient 11). Following reduction under sedation he had no further symptoms (constant score 84% of control). He had voluntarily stopped playing sport at the time of review but was still satisfied with surgery as his symptoms were much better than pre-operatively. The second patient had a further dislocation with minimal trauma (patient 10). He was able to relocate his shoulder himself and again was stable at the time of review (constant score 78% of control). One of the unsatisfied patients had significant symptomatic instability, had the lowest constant score that we measured (67% of control), and did not return to pre-injury level of activity (patient 2). The other two had lower constant scores than average (69% and 84% of control) but had no recurrence of instability or dislocation (patients 12 and 20). Neither, however, was able to return to their desired activities. One had to change profession and the other gave up competitive rugby. Although expectations of surgery were not formally recorded, all these three patients, whilst accepting there was a small failure rate, assumed that surgery would allow them to return to normal. When assessing any medical intervention, surgical or otherwise, patient’s satisfaction outweighs any other endpoint. In the young, active patient, return to previous activity is often the most important factor. Although undoubtedly linked it must be remembered, as illustrated by our findings, that satisfaction and recurrence of symptoms do not always coincide. We believe patients should be warned of an approximately 10-15% failure rate and that their shoulders are likely to return to 80-90% of normal. This may preclude them from high risk occupations and sports in up to 1/3 of cases. This should be emphasized in the younger patient as all of our recurrences were aged 25 or less.

The range of motion following open surgery is usually reduced. The greater soft tissue dissection (when compared with arthroscopy) and division/repair of subscapularis lead to loss of external rotation (ER), particularly when combined with abduction. However, it is precisely this movement that causes the greatest amount of apprehension when examining the patient with anterior shoulder instability. It has been suggested that by reducing the amount of external rotation, the patient no longer enters their “apprehension zone” and therefore no longer suffers with symptomatic instability. One study comparing transglenoid sutures with open repair found that 45% of patients in the open group lost some ER (10-40°) but there were no re-dislocations. The arthroscopic group had no loss of motion but a 60% recurrence of instability, suggesting that reducing the range of motion reduces recurrence.[15]

Both imbrication of redundant capsule and closure of the rotator interval have been recommended in addition to repair of the Bankart lesion and intuitively are both likely to reduce the overall range of motion.[16] Even using new arthroscopic anchors, ER in abduction can be reduced by up to 14°.[17] This is contradicted however by a recent study looking at knotless and bioknotless anchors showing 1° loss of ER with only 7% recurrence of symptoms.[10] Our study also found very little reduction in the range of motion following the same procedure with an average loss of ER in abduction of 4° ± 4°. Although measurement was very accurate it was limited to only four patients.

Qualitatively, the graphs showing range of motion over time show a much smoother velocity of shoulder movement post-stabilization [Figure 3]. Pre-operatively there is an interruption in the smooth motion from neutral to full ER. We believe that this interruption or “catch” is due to a protective muscular contraction as the patient enters their apprehension zone. Over time, they are able to voluntarily overcome this until their limit it reached. In the example shown, this “catch” occurs at 52° and the ultimate range of ER in abduction is 74°. Post-operatively the curve is much smoother and is closer to that of a normal individual, where a smooth velocity of shoulder movement throughout internal and external rotations is observed. In our example, the total range of ER in abduction was 79°. Fastrack analysis in this instance provides some evidence to support the ability of the bioknotless anchor to provide a stable shoulder whilst maintaining range of motion. The patient moves through their apprehension zone without interruption to a slightly better limit than pre-operatively. This contradicts the theory that shoulder stabilization works by reducing the range of ER.

## CONCLUSIONS

Our results are broadly in line with others reporting their experience with the bioknotless anchor. The fastrack analysis demonstrates that patients who have a successful outcome have on average, a slight decrease of external rotation, but this is not as significant as that seen in open stabilization. The velocity of the external rotation arc is smoother post-operatively and the interruption or “catch” seen during preoperative analysis is abolished. Our study suggests that arthroscopic stabilization is an effective procedure which restores stability without significantly decreasing range of motion, contrary to the results seen with open stabilization.
